# The upregulation of *CLGN* in hepatocellular carcinoma is potentially regulated by hsa-miR-194-3p and associated with patient progression

**DOI:** 10.3389/fonc.2022.1081510

**Published:** 2023-01-09

**Authors:** Zhongyuan Cui, Jielong Wang, Gang Chen, Dongliang Li, Bianqiao Cheng, Yanhua Lai, Zhixian Wu

**Affiliations:** ^1^ Department of Hepatobiliary Disease, Dongfang Hospital, School of Medicine, Xiamen University, Fuzhou, China; ^2^ Department of Gastroenterology, Liuzhou Workers’ Hospital (The Fourth Affiliated Hospital), Guangxi Medical University, Liuzhou, China; ^3^ Department of Gastroenterology, Fuzhou Second Hospital, Fuzhou, China; ^4^ Department of Transplantation, People’s Hospital of Guangxi Zhuang Autonomous Region, Guangxi, China

**Keywords:** hepatocellular carcinoma (HCC), prognosis, CLGN, MND1, STXBP6, MiR-194-3p

## Abstract

**Background:**

Patients with hepatocellular carcinoma (HCC) have poor prognosis, especially in advanced stages. Targeted therapy is the main treatment for advanced HCC patients, but the optimal targets for HCC remain poorly understood. The main purpose of this study was to identify potential novel prognostic markers and therapeutic targets.

**Methods:**

Firstly, differentially expressed genes (DEGs) in HCC were identified from the Gene Expression Omnibus (GEO) database. The expression, significance in prognosis, and potential mechanisms of DEGs were analyzed using GEPIA, TIMER, HPA, Kaplan Meier Plotter, CBioPortal, miRWalk, TargetScan, and ENCORI databases. Immunohistochemical staining was used to determine the protein expression levels of potential candidate genes.

**Results:**

The mRNA levels of *MND1*, *STXBP6*, and *CLGN* were significantly increased in HCC (*p*< 0.01). HCC patients with elevated *CLGN* mRNA levels had poorer overall survival (OS), disease-free survival (DFS), progression-free survival (PFS), and disease-specific survival (DSS) (*p* < 0.05). Higher *MND1* mRNA levels significantly correlated with poorer DFS in HCC patients (*p*< 0.05). However, there was no significant correlation between *STXBP6* expression and prognosis of HCC (*p*> 0.05). Further analysis revealed that patients with elevated *CLGN* mRNA expression in advanced pathology stages had poorer prognosis (*p*< 0.01). In addition, CLGN protein levels were elevated in HCC compared to their levels in normal tissues. The mRNA levels of *CLGN* had no significant correlation with the abundance of six common tumor infiltrating lymphocytes in HCC (COR < 0.5). Moreover, the mutation rate of *CLGN* was less than 1% in HCC patients (10/1089). Finally, the expression level of hsa-miR-194-3p in HCC was significantly lower than that in normal tissues (*p* < 0.05), and prognosis of HCC with low expression of hsa-miR-194 was poor (*p* < 0.05).

**Conclusion:**

The upregulation of *CLGN* in HCC is significantly associated with poor patient prognosis, especially in the advanced stages, and may be regulated by hsa-miR-194-3p. These findings suggest that *CLGN* may be closely related to the progression of HCC, and is a potential therapeutic target and prognostic indicator for patients with advanced HCC.

## Introduction

Primary liver cancer remains the sixth most common cancer and the third leading cause of cancer-related deaths worldwide in 2020, according to the latest statistics (1). Hepatocellular carcinoma (HCC) encompasses 75% -85% of liver cancer cases (1). Most HCC is in advanced stages at the time of diagnosis and is resistant to conventional cytotoxic drugs. Currently, systemic therapy for patients with advanced HCC includes molecularly targeted agents, immune checkpoint inhibitors, or a combination of both (2–4). However, a substantial proportion of patients have yet to benefit from systemic therapy (5, 6). Recent studies have identified several novel biomarkers that can predict HCC prognosis. Lu et al. showed that serum α-fetoprotein trajectories were associated with overall survival of patients with intermediate-stage HCC after transarterial chemoembolization (7). However, it is imperative to identify the most effective predictive biomarkers to facilitate individualized and targeted treatments. Therefore, it is necessary to screen more biomarkers to improve patient diagnosis, and prognosis and identify therapeutic targets for precision therapy.

In recent years, bioinformatic databases have provided abundant transcriptomic and clinical data (8, 9), which are convenient for the analysis of relevant markers with potential significance (10–12). In this study, we explored several databases for molecular markers of HCC and investigated their expression levels in clinical specimens.

## Methods

### Screening of DEGs in HCC patients

Tumor and normal tissue Gene expression datasets GSE54236 (https://www.ncbi.nlm.nih.gov/geo/query/acc.cgi?acc=GSE54236) (13) and GSE121248 (https://www.ncbi.nlm.nih.gov/geo/query/acc.cgi?acc=GSE121248) were downloaded from Gene Expression Omnibus (GEO, http://www.ncbi.nlm.nih.gov/geo/) (14). D ifferentially expressed genes (DEGs) were analyzed using the online tool GEO2R. Gene Expression Profiling Interactive Analysis (GEPIA, http://gepia.cancer-pku.cn/) (15) and Tumor Immune Estimation Resource database (TIMER, https://cistrome.shinyapps.io/timer/) (16) were used to further verify the DEGs. A *p-*value of <0.05 was statistically significant.

### The significance of DEGs in the prognosis of HCC

GEPIA was used to analyze the relationship between DEGs and overall survival (OS) and disease-free survival (DFS) in HCC patients. Kaplan Meier Plotter (http://kmplot.com/analysis/index.php?p=service&cancer=liver_rnaseq) (17) was used to verify the relationship between candidate gene expression levels and OS, progression-free survival (PFS), relapse-free survival (RFS), and disease-specific survival (DSS) in HCC patients. Then, Kaplan Meier Plotter was used to explore the relationship between candidate genes and OS in patients with different pathological stages. Patients were trichotomized (T1 vs. T3).

### CLGN protein expression in HCC and normal tissues

Ethics Statement: The study was conducted in accordance with the principles of the Declaration of Helsinki (18). The collection of HCC specimens was approved by the Ethics Committee of Liuzhou Workers’ Hospital (No.KY2022051).

Immunohistochemical Staining: Samples from 24 HCC patients who underwent surgical resection at Liuzhou Workers’ Hospital were collected. The acquisition of specimens was approved by the Ethics Committee of Liuzhou Workers’ Hospital.

Five-micrometer sections were obtained from paraffin-embedded tumor and non-tumor specimens. All sections were dewaxed in xylene and rehydrated in alcohol, followed by wet autoclave pretreatment in citrate buffer for antigen retrieval (10 min at 120°C, pH=6.0) and then rinsed with phosphate buffer saline. Immunohistochemical staining with antibody to *CLGN* (Anti-Calmegin/CLGN Antibody, Rabbit: A05261-1, Boster Biological Technology Co. Ltd.) was performed using the avidin-biotin- peroxidase complex method. The primary antibody (1:200 dilution for *CLGN*) was applied to the sections and allowed to bind for 1 h at room temperature. The sections were then incubated with biotinylated anti-mouse/rabbit antibody for 30 min and an avidin-biotin-peroxidase reagent for 10 min. After color development with diaminobenzidine, the sections were counterstained with hematoxylin.

In addition, PATHOLOGY module in HPA (https://www.proteinatlas.org/) database was used to verify the expression of CLGN protein in HCC patient specimens and normal liver tissue.

### Analysis of mutation and immune cell infiltration levels

The CBioportal database (https://www.cbioportal.org/) (19) was used to analyze the mutation of *CLGN* in HCC patients. The relationships between the expression levels of *CLGN* and six immune cells (B cells, CD4+ T cells, CD8+ T cells, neutrophils, macrophages, and dendritic cells) in HCC were estimated using the TIMER algorithm. Statistical significance was set at *p* values of <0.05 and correlation coefficients (COR) of ≥ 0.5.

### Screening of regulating microRNAs

MiRWalk (http://mirwalk.umm.uni-heidelberg.de/) (20) and TargetScan (http://www.targetscan.org/vert_72) (21) databases were used to predict microRNAs (miRNAs) that regulate *CLGN* mRNA expression. The Encyclopedia of RNA Interactomes (ENCORI, http://rna.sysu.edu.cn/encori/mirTarPathways.php) (22) database was used to analyze the expression levels of candidate miRNAs in HCC and normal tissues. Kaplan Meier Plotter was used to analyze the relationship between the levels of candidate miRNAs and prognosis of HCC. A *p* value of <0.05 was considered statistically significant.

### Data processing and visualization

Gene expression data were cleaned and visualized using R (Version 3.6.3) and the R packages ggplot2 and VennDiagram. The cleaning algorithm for the gene expression data included removal of non-coding genes, miRNA, and blank probes; FDR ≤0.05 and FC ≥2; retaining one level from one gene that had several high or, consistently, low expression levels in different specimens. We removed genes that had both high expression and low expression in different specimens. Cytoscape (Version: 3.7.2) and cytoHubba plugin were used to analyze miRNAs with the potential to regulate *CLGN* mRNA expression.

## Results

### Significant DEGs in HCC

**Figure 1:**
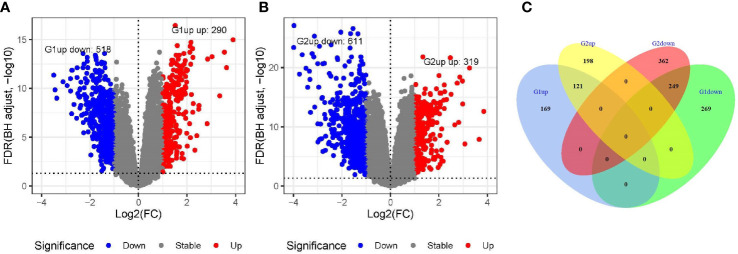
Screening of differentially expressed genes in GSE54236 (G1) and GSE121248 (G2) datasets. **(A, B)** Gene expression levels in GSE54236 (G1) and GSE121248 (G2) datasets, with blue dots indicating significantly low expression and red dots indicating significantly high expression. **(C)** The number of genes with high or low expression in both datasets.

The DEG analysis revealed 290 genes with significantly high expression and 518 genes with significantly low expression in the GSE54236 (G1) dataset (**Figures 1A, C**). In the GSE121248 (G2) dataset, 319 genes were significantly upregulated, and 611 genes were significantly under-expressed (**Figures 1B, C**). There were 121 genes with high expression and 249 genes with low expression in both the datasets (**Figure 1C**). After removing the genes that have been reported, we found that the expression levels of *MND1*, *STXBP6*, and *CLGN* in HCC were significantly higher than those in normal tissues.

**Figure 2:**
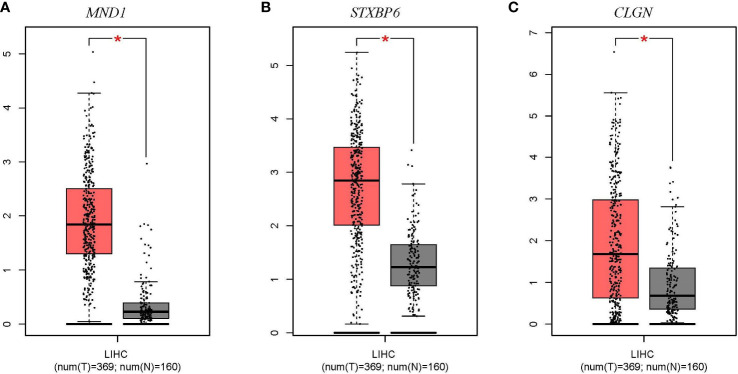
The mRNA expression levels of *MND1*, *STXBP6*, and *CLGN* in HCC (GEPIA). **(A-C)** The mRNA expression levels of *MND1*, *STXBP6*, and *CLGN* in HCC were significantly higher than in normal tissue. *p < 0.01.

**Figure 3:**
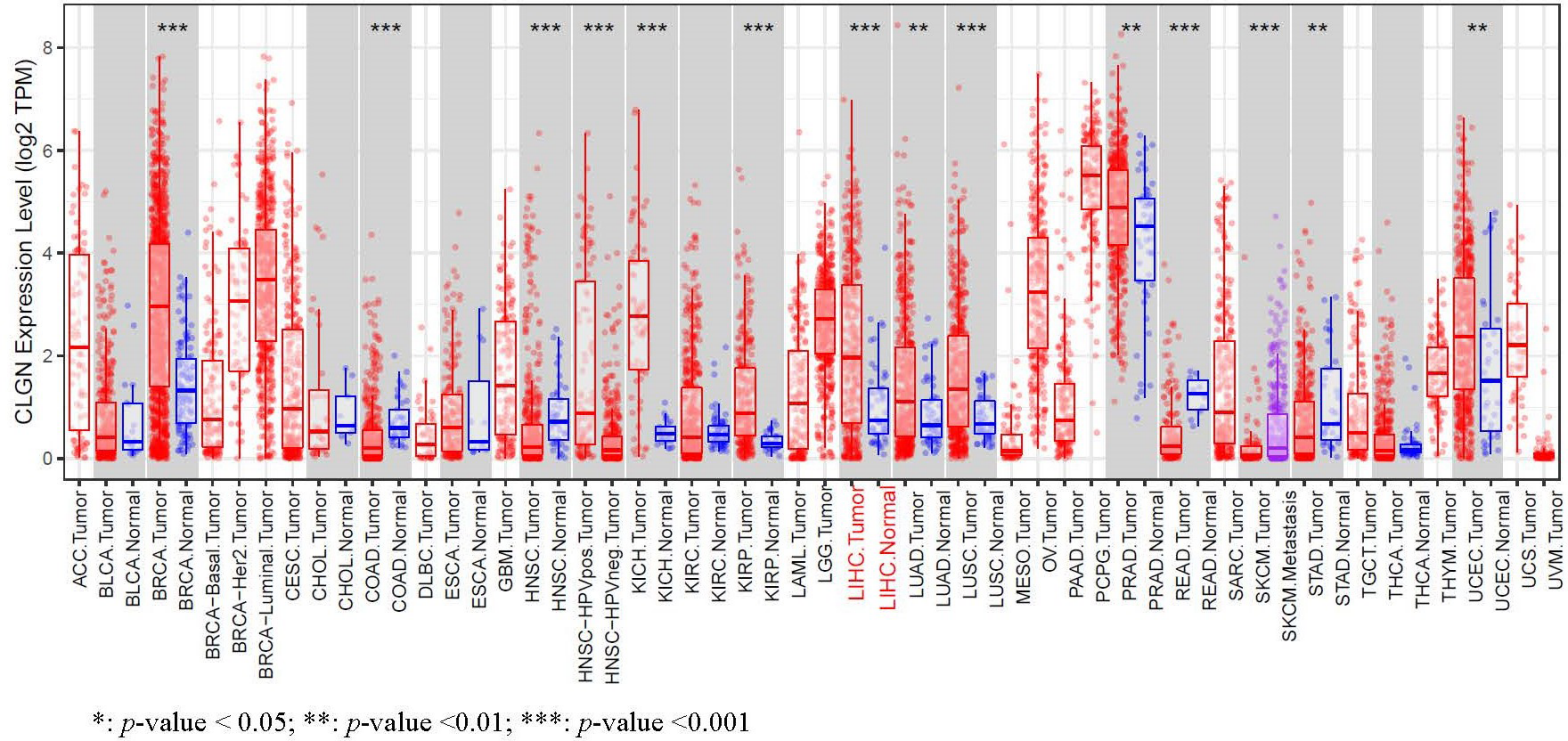
The mRNA expression of levels of *CLGN* in several common human tumors. The mRNA of *CLGN* was upregulated in liver and other tumors (TIMER).

The expression levels of *MND1*, *STXBP6*, and *CLGN* mRNA in HCC w ere verified. The results showed that the *MND1*, *STXBP6*, and *CLGN* mRNA levels in HCC were significantly higher than those in normal tissues (*p* < 0.01, **Figures 2A-C**). In addition, the mRNA expression of *CLGN* was high in breast invasive carcinoma, kidney chromophobe, kidney renal papillary cell carcinoma, lung adenocarcinoma, lung squamous cell carcinoma prostate adenocarcinoma, and uterine corpus endometrial carcinoma (*p* < 0.01, **Figure 3**).

### Relationship between DEGs and prognosis of HCC

**Figure 4:**
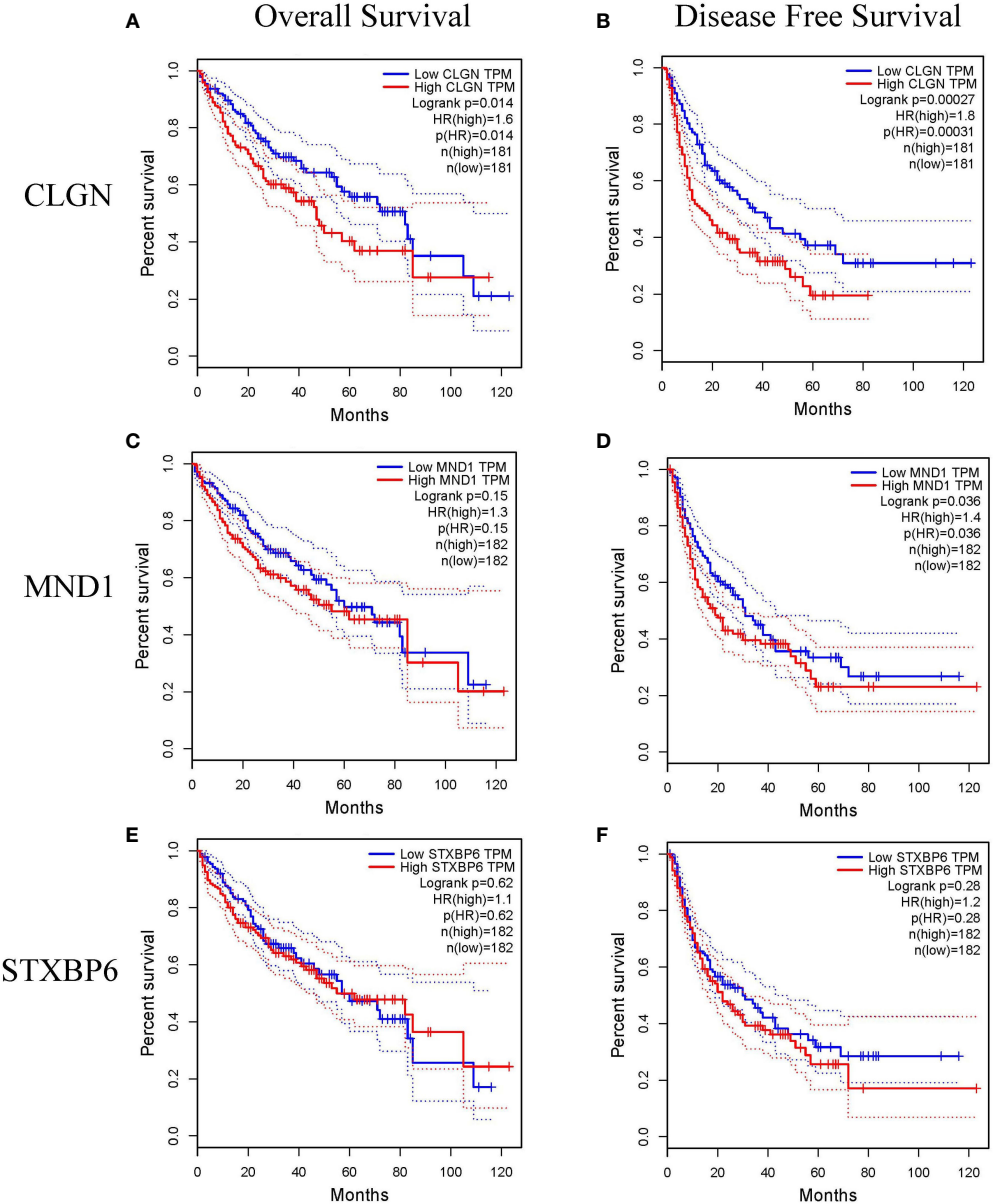
The relationship between the mRNA levels of *MND1*, *STXBP6* and *CLGN* and the prognosis of HCC (GEPIA). **(A, B)** HCC patients with higher mRNA levels of *CLGN* had poorer overall survival and disease-free survival (*p*< 0.05). **(C)** No significant relationship between *MND1* mRNA levels and patients’ overall survival was observed (*p* > 0.05). **(D)** Patients with higher *MND1* mRNA levels had poorer disease-free survival (*p* < 0.05). **(E, F)** There was no significant correlation between the mRNA levels of *STXBP6* and overall survival and disease-free survival in HCC patients (*p* > 0.05).

The prognostic value of *MND1*, *STXBP6*, and *CLGN* in HCC patients was explored. HCC patients with higher mRNA levels of *CLGN* had poorer OS and DFS (*p*< 0.05, **Figures 4A, B**). There was no significant correlation between *MND1* mRNA levels and OS (*p*> 0.05; **Figure 4C**), and patients with higher *MND1* mRNA levels had poorer DFS (*p*< 0.05, **Figure 4D**). However, there was no significant correlation between the mRNA levels of *STXBP6* and OS or DFS in HCC patients (*p* > 0.05, **Figures 4E, F**).

**Figure 5:**
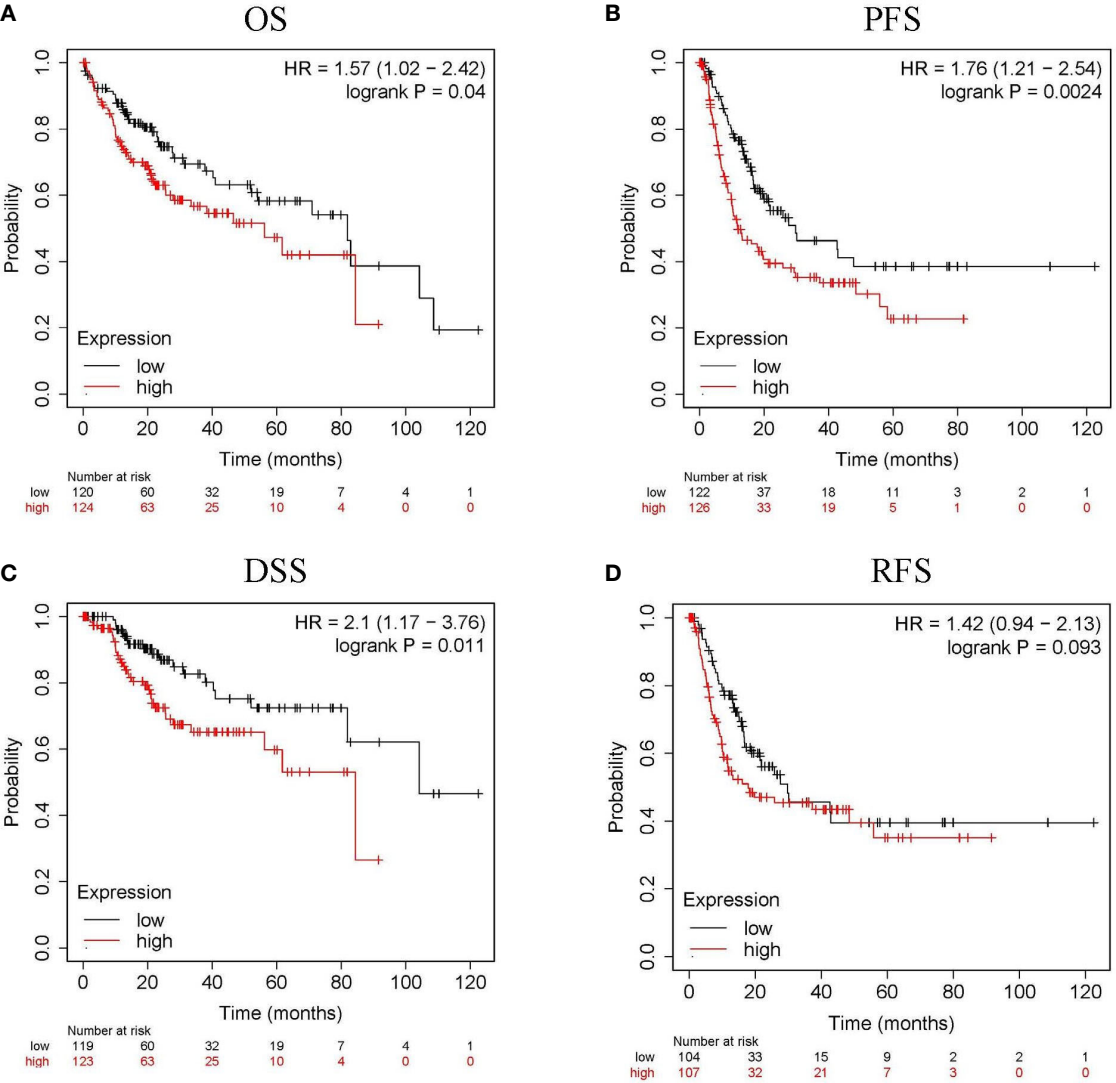
The relationship between *CLGN* mRNA and prognosis of HCC (Kaplan Meier Plotter). **(A–C)** HCC patients with higher *CLGN* mRNA expression have poorer OS, PFS, and DSS, *p* < 0.05. **(D)** There was no significant relationship between *CLGN* mRNA level and RFS, *p* > 0.05.

**Figure 6:**
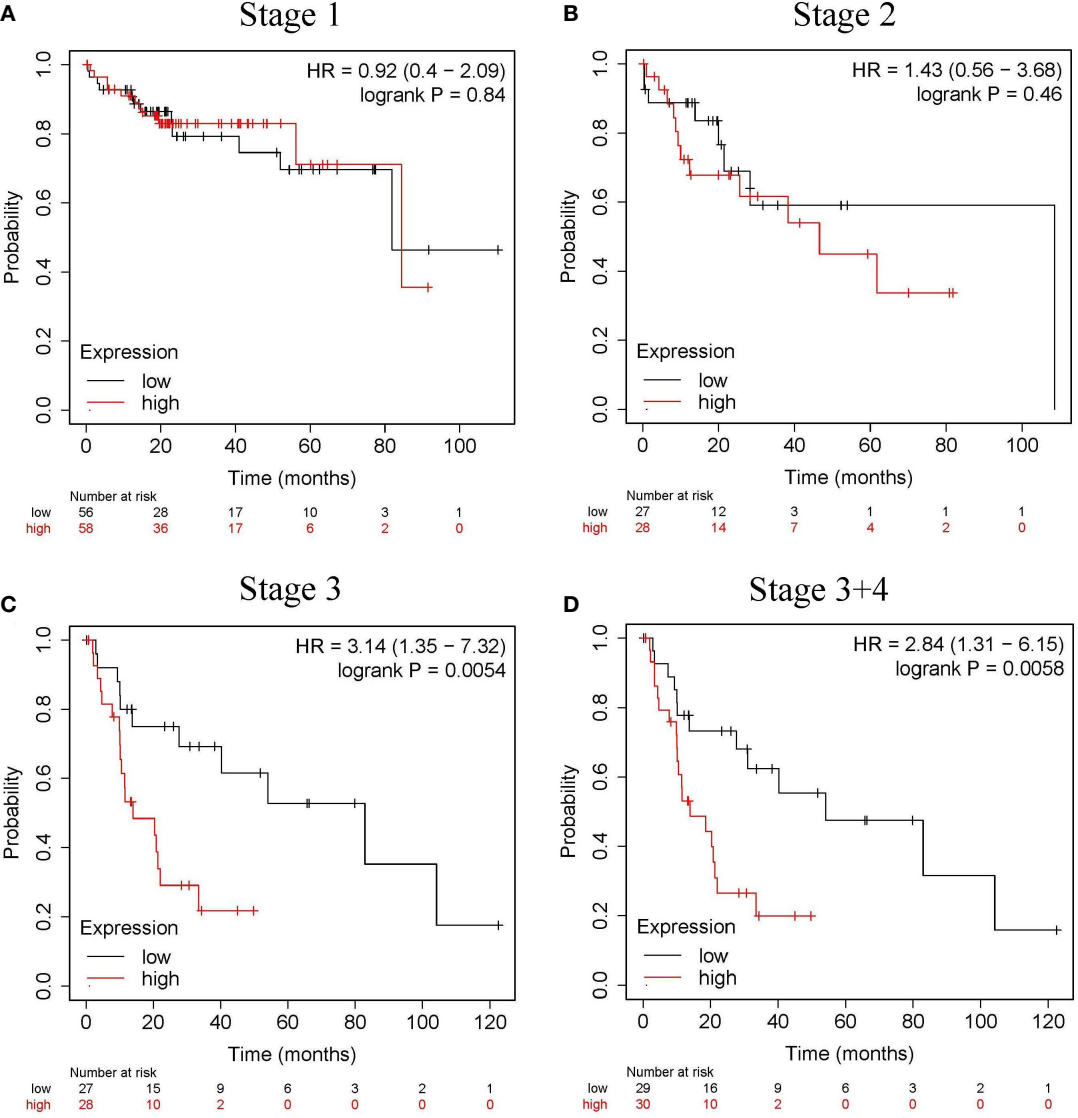
The relationship between the *CLGN* mRNA level and the OS in different pathology stages in HCC patients. (Kaplan Meier Plotter). **(A, B)** There was no significant correlation between the *CLGN* mRNA level and the OS in HCC patients in early stages (T1 and T2), *p*> 0.05. **(C, D)** HCC patients in advanced T stages (T3 and T3+T4) have higher *CLGN* mRNA expression and poorer OS, *p* < 0.05.

The relationship between *CLGN* mRNA levels and the prognosis of HCC was also investigated. The results showed that the mRNA levels of *CLGN* were significantly correlated with OS, PFS, and DSS in HCC patients (*p*< 0.05, **Figures 5A-C** and **Table 1**). However, there was no significant relationship between *CLGN* mRNA levels and RFS (*p*> 0.05, **Figure 5D** and **Table 1**). Furthermore, the mRNA level of *CLGN* was significantly correlated with the OS in different pathology stages in HCC patients. Specifically, HCC patients with higher mRNA expression of *CLGN* in advanced stages (T3, T3+T4) had poorer OS (*p* < 0.01, **Figures 6C, D** and **Table 2**). No significant correlation was found between *CLGN* mRNA levels and OS in patients with early-stage HCC (T1-T2) (*p* > 0.05, **Figures 6A, B** and **Table 2**).

**Table 1:** Correlation between the *CLGN* mRNA expression and the survival of HCC patients.

Gene	Survival	Patients	HR	*p* Value	Median survival (months)	
					Low	High
*CLGN*	OS	244	1.57	0.0401	81.9	56.2
	RFS	211	1.42	0.0929	29.77	17.9
	PFS	248	1.76	0.0024	29.77	11.83
	DSS	242	2.10	0.0106	104.17	84.4

**Table 2:** Correlation between the *CLGN* mRNA expression and patient OS at different pathological stages.

Stage	Patients	HR	*p* Value	Median survival (months)	
				Low	Low
1	170	0.92	0.84	81.9	84.4
2	83	1.43	0.46	108.6	46.6
1+2	253	1.35	0.34	108.6	84.4
3	83	3.14	0.0054	82.9	14
3+4	87	2.84	0.0058	54.1	14

### CLGN protein expression in clinical specimens

**Figure 7:**
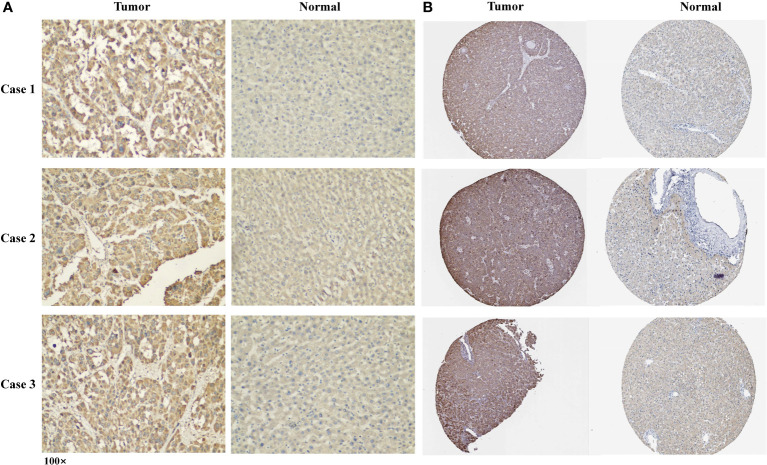
The expression of CLGN protein in HCC and adjacent tissues. **(A)** Three typical cases of CLGN protein upregulation in HCC in 24 clinical specimens. **(B)** The expression of CLGN protein in HCC is higher than non-cancerous liver tissue (HPA).

The p rotein expression of CLGN in tumor and normal tissues of clinical specimens from patients with HCC was investigated. Immunohistochemical staining showed that the CLGN protein was significantly regulated in HCC compared with normal liver tissues in 58% (14/24) of cases (**Figure 7A**). The HPA database results also showed that CLGN protein levels were higher in HCC than in non-tumor tissues (**Figure 7B**).

### *CLGN* mutation in HCC and the correlation between *CLGN* expression and tumor infiltrating lymphocytes

**Figure 8:**
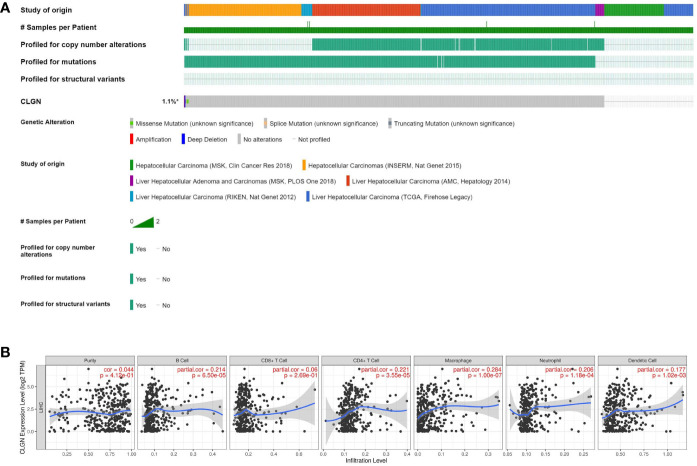
*CLGN* mutations in HCC patients and the correlation between *CLGN* expression and tumor infiltrating lymphocytes. **(A)** *CLGN* mutations occurred in 10 (< 1%) of 1089 patients from six data sources (CBioportal). **(B)** There is no significant correlation between the mRNA of *CLGN* and the infiltrating levels of B cells, CD4+ T cells, CD8+ T cells, neutrophils, macrophages, and dendritic cells in HCC, COR < 0.5 (TIMER).

*CLGN* mutations were analyzed in 1089 patients with HCC from six data sources. The results showed that *CLGN* was mutated in < 1% (10/1089) of patients (**Figure 8A**). Furthermore, there was no significant correlation between *CLGN* expression and the levels of B cells, CD4+ T cells, CD8+ T cells, neutrophils, macrophages, or dendritic cells in HCC (COR < 0.5 (**Figure 8B**).

### miRNAs that regulate *CLGN*

**Figure 9:**
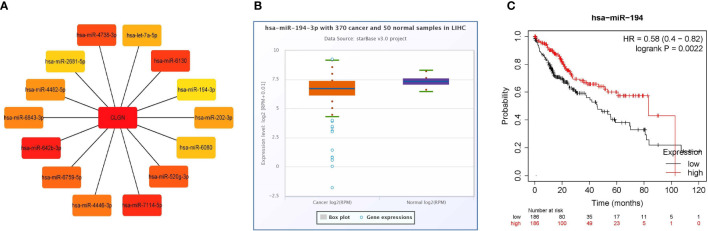
miRNAs that likely regulate the expression of *CLGN* mRNA (miRWalk and TargetScan). **(A)** The 14 miRNAs that most closely related to *CLGN* mRNA. **(B)** The expression of hsa-miR-194-3p in HCC is significantly lower than in normal tissue, *p* < 0.05 (ENCORI). **(C)** HCC patients with lower hsa-miR-194 expression have poorer OS than those without, *p* < 0.05 (Kaplan Meier Plotter).

Firstly, 4357 and 634 miRNAs that potentially regulate *CLGN* were predicted in the miRWalk and TargetScan databases, respectively. Duplicate data from the two groups were removed, and their intersection contained 248 potential miRNAs (**Supplementary 1**). As calculated with Cytoscape, the top 14 miRNAs that were most likely to regulate *CLGN* mRNA expression were hsa-miR-642b-3p, hsa-miR-7114-5p, hsa-miR-6130, hsa-miR-4738-3p, hsa-miR-520g-3p, hsa-miR-6759-5p, hsa-miR-4446-3p, hsa-miR-6843-3p, hsa-miR-4482-5p, hsa-miR-202-3p, hsa-let-7a-5p, hsa-miR-6080, hsa-miR-2681-5p, and hsa-miR-194-3p (**Figure 9A**). A further analysis revealed that hsa-miR-194-3p was significantly underexpressed in HCC (Fold Change = 0.72, *p* < 2.1e-5 and FDR< 0.001, **Figure 9B**). HCC patients with low hsa-miR-194 expression had poorer OS than those without (*p* < 0.001, **Figure 9C**).

## Discussion

CLGN (Calmegin) is a testis-specific endoplasmic reticulum chaperone protein (23, 24). Previous studies have shown that CLGN acted as a chaperone for one or more sperm surface proteins to mediate the interaction between sperm and eggs during spermatogenesis (23). Therefore, *CLGN* may play an important role in spermatogenesis and infertility. A recent study found that *CLGN* was up-regulated in aldogen-producing adenomas and was related to the generation of aldosterone (25). However, the role of *CLGN* in several malignant tumors, including HCC, remains unclear.

In this study, it was found that the mRNA and protein of *CLGN* were highly expressed in HCC compared with normal liver tissues. Furthermore, patients with higher *CLGN* mRNA levels had poorer prognoses, and *CLGN* mRNA levels were valuable in predicting the prognosis of patients with advanced pathology. These results suggest that *CLGN* is associated with malignant progression in HCC patients. Additionally, *CLGN* was found to be significantly upregulated in invasive breast cancer, chromophobe renal cell carcinoma, papillary renal cell carcinoma lung adenocarcinoma, lung squamous cell carcinoma, prostate adenocarcinoma, and uterine corpus endometrial carcinoma. To the best of our knowledge, no studies have investigated the role of *CLGN* in these tumors, which is worth of further investigation.

However, the relationship between *MND1* and STXBP6 in HCC remains unknown. Notably, Zierhut et al. found that *MND1* was necessary for meiosis homologue repair (26). Zhang et al. found that *MND1* was upregulated in lung adenocarcinoma and was an independent risk factor for overall survival (27). Furthermore, *MND1* was a prognostic biomarker for renal clear cell carcinoma (28). Lenka et al. found that *STXBP6* hypermethylation was associated with adverse clinical outcomes in patients with lung cancer (29). A nother recent study showed that the expression level of *STXBP6* predicted the response to PD-1/PD-L1 immunotherapy in patients with cancer (30). In this study, we found that *MND1* and *STXBP6* were upregulated in HCC, but no significant correlation was found between their expression and patient prognosis .

Gene mutations are a common tumorigenic mechanism in liver cancer (31–34). In this study, it was found that *CLGN* was rarely mutated in HCC patients, which suggests that the pathological effects of *CLGN* may not be exerted through genetic mutations. The immune microenvironment is closely related to tumor progression (35–37). Therefore, we also analyzed the relationship between *CLGN* mRNA levels and the abundance of tumor-infiltrating lymphocytes in HCC; however, no significant correlation was found. Based on this, we hypothesized that *CLGN* might influence the progression of HCC by promoting cell proliferation or inhibiting tumor cell apoptosis, which requires further study in the future.

MicroRNAs (miRNAs) are small non-coding RNAs composed of approximately 20 nucleotides that regulate gene expression by binding to the 3’-UTR of the target mRNA (38). Recent studies have shown that miRNAs were an important regulator of mRNA expression (39–41). hsa-miR-194-3p was significantly underexpressed in HCC, and patients with lower hsa-miR-194 expression had poorer prognosis. A study found that hsa-miR-194-3p might target MECP2 to promote breast cancer progression and reduce sensitivity to rapamycin (42). Recent studies have shown that hsa-miR-194-3p might be associated with colorectal cancer progression (43). However, its specific therapeutic and prognostic value in tumors requires further investigation. In this study, we discovered that hsa-miR-194-3p may also play an important role in HCC progression by regulating *CLGN* expression.

Our findings suggest that *CLGN* is a potential prognostic marker for HCC and is associated with HCC progression, however, several limitations are associated with our study. Specifically, the relationship between *CLGN* expression and patient prognosis has not yet been verified at the center. Additionally, laboratory research on the cancer-promoting mechanisms of *CLGN* were not available, and the sample size used for immunohistochemistry in this study was small. Therefore, a large-scale verification study is required in the future.

## Conclusion

*CLGN* is upregulated in HCC and significantly correlates with patient prognosis, especially in the advanced stages. In addition, the mRNA levels of *CLGN* were found to be potentially regulated by hsa-miR-194-3p. These results suggest that *CLGN* may have important therapeutic and prognostic value in HCC patients.

## Data availability statement

The datasets analyzed during the current study are available on Gene Expression Omnibus (GEO) (accession numbers: GSE54236, GSE121248), Gene Expression Profiling Interactive Analysis (GEPIA), Tumor Immune Estimation Resource (TIMER), Kaplan Meier Plotter, The Human Protein Atlas (HPA), cBioPortal, miRWalk, TargetScan, and The Encyclopedia of RNA Interactomes (ENCORI).

## Ethics statement

The studies involving human participants were reviewed and approved by Ethics Committee of Liuzhou Workers’ Hospital. Written informed consent for participation was not required for this study in accordance with the national legislation and the institutional requirements.

## Author contributions

ZC: Data analysis and writing of the first draft. BC, YL and ZW: Data analysis, manuscript editing, review, and approval. JW, ZC, and GC: Data analysis, data collection and experiments. DL: Data analysis, manuscript editing and review. All authors have read and approved the final manuscript.
